# ﻿After a decade, a new Venezuelan species of *Corydalus* Latreille (Megaloptera, Corydalidae, Corydalinae) is discovered

**DOI:** 10.3897/zookeys.1111.76884

**Published:** 2022-07-11

**Authors:** Caleb Califre Martins, Carlos A. S. de Azevêdo, Neusa Hamada, Maria E. Grillet, Atilano Contreras-Ramos

**Affiliations:** 1 Instituto de Biología, Departamento de Zoología, Colección Nacional de Insectos, Universidad Nacional Autónoma de México, 04510 Mexico City, Mexico Universidad Nacional Autónoma de México Mexico City Mexico; 2 Laboratório de Entomologia Aquática, Centro de Estudos Superiores de Caxias, Universidade Estadual do Maranhão, 65604380 Caxias, Maranhão, Brazil Universidade Estadual do Maranhão Maranhão Brazil; 3 Coordenação de Biodiversidade, Instituto Nacional de Pesquisas da Amazônia-INPA, 69067–375 Manaus, Amazonas, Brazil Instituto Nacional de Pesquisas da Amazônia Manaus Brazil; 4 Laboratorio de Biología de Vectores y Parásitos, Instituto de Zoología y Ecología Tropical, Facultad de Ciencias, Universidad Central de Venezuela, Apartado 47058, Caracas 1041-A, Venezuela Universidad Central de Venezuela Caracas Venezuela

**Keywords:** Aquatic insects, biodiversity, Corydalinae, dobsonfly, Neotropics, taxonomy

## Abstract

A new species of dobsonfly from Venezuela, *Corydalusralphi* Martins, Azevêdo, Hamada & Contreras, **sp. nov.**, was discovered a decade after the last description of a species of this genus in the country. The new species is morphologically similar to *C.wanningeri* Contreras-Ramos & von der Dunk, sharing a uniform reddish coloration of body and wings and similar male genitalic structures. Likewise, it shares this particular coloration with *C.neblinensis* Contreras-Ramos but the genitalic structure fits within the *C.crossi* Contreras-Ramos species group. Two specimens, one male and one female, were collected on Tarotá River, in the Gran Sabana region, Canaima National Park, in southern Venezuela. A key to identify males of the Venezuelan species of *Corydalus* is provided.

## ﻿Introduction

*Corydalus* Latreille is the most species rich genus of dobsonflies from the New World. This genus was revised 23 years ago by Contreras-Ramos (1998), and since then several more species have been described (Contreras-Ramos 2002; Contreras-Ramos and von der Dunk 2010; Ardila-Camacho 2014; Ardila-Camacho and Contreras-Ramos 2018) adding up to 39 extant valid species of *Corydalus*, plus one doubtful species that occurs in Indonesia: *Corydalustestaceus* Le Peletier de Saint Fargeau & Audinet-Serville in Latreille et al. 1828. Of the 39 valid species, 34 occur only in the Neotropical region, one species is restricted to the Nearctic region, and three species occur in both regions (Oswald 2021).

In total, 33 species of *Corydalus* occur in South America (Oswald 2021). Venezuela is the South American country with the greatest diversity of this genus, with 16 species described to date. Brazil is the second one with 13 species, followed by Colombia with 12 species. Recently, we studied specimens from Instituto Nacional de Pesquisas da Amazônia (INPA) which were temporarily on loan at Instituto de Biología-UNAM, and we found a couple of specimens from the Gran Sabana region, Canaima National Park Parque Nacional, Bolívar state, Venezuela that belong to an undescribed species, the 17^th^ from this country.

*Corydalusralphi* sp. nov. is superficially similar to *C.neblinensis* Contreras-Ramos (e.g., similar color of body and wings), yet it appears most closely related to *C.wanningeri* Contreras-Ramos & von der Dunk, both fitting within the *C.crossi* Contreras-Ramos species group. All these species are from Venezuela, the latter two described from Bolivar state. We are glad to make this contribution as part of a highly deserved homage to Prof. Ralph W. Holzenthal, who has studied Neotropical insect biodiversity, especially Trichoptera, and guided a large number of students for more than three decades.

## ﻿Materials and methods

Several larvae of *Corydalus* were collected in Venezuela by Carlos Augusto Silva de Azevêdo and Neusa Hamada in the year 2007 by manual method; larvae were placed in containers with local ground substrate, so that several pupated and adults emerged, including two of the new species. This material was sent to ACR a few years ago, but was only recently examined. Specimens were collected on the Río Tarotá (Tarotá River), located on Canaima National Park, within the Gran Sabana region, Bolívar state, southern Venezuela. This region is composed by an upland savanna covering close to 18,000 km^2^, with altitudes ranging from 750 to 1,450 m a.s.l., with a humid submontane climate, with average annual temperature ranging between 18 °C and 24 °C, and average annual rainfall between 2,000 and 3,000 mm (Huber 1995). This area is drained by tributaries of the Orinoco River, most of them black-water rivers, with very acidic and low mineral waters such as the Tarotá River (Huber 1995). To study genital structures, abdomen was cut between 7^th^ and 8^th^ segments, then cleared in 10% potassium hydroxide (KOH) overnight at room temperature, rinsed with distilled water, observed in 80% ethyl alcohol, and posteriorly stored in microvials with glycerin, each pinned below the respective specimen. Observation of the genitalic morphology was made in Petri dishes below a Zeiss Discovery V8 stereomicroscope.

Drawings were made using a drawing tube attached to a stereomicroscope, and then they were vectorized using the program Adobe Illustrator CS6. Series of images of different focus were made using an Olympus TG-4 camera attached to a manual copy stand, posteriorly they were combined using the software HeliconFocus 6.7.1. Drawings and images were edited using the software Adobe Photoshop CS6. A distribution map was produced with the website http://www.simplemappr.net. Morphological terminology follows New and Theischinger (1993) for general morphology, Liu et al. (2016) for genital sclerites, and Breitkreuz et al. (2017) for wing venation. Specimens will be deposited at the Entomological Collection of the Instituto Nacional de Pesquisas da Amazônia (**INPA**), Manaus, Amazonas, Brazil.

## ﻿Taxonomy

### ﻿ Corydalus ralphi

Taxon classificationAnimaliaMegalopteraCorydalidae

Martins, Azevêdo, Hamada & Contreras sp. nov.

27510E0B-009F-57B5-BDE9-B8DD1636A705

http://zoobank.org/5AFD72F9-1AB7-44B1-BB65-BABB9E1F2E1D

**Figure 1.**
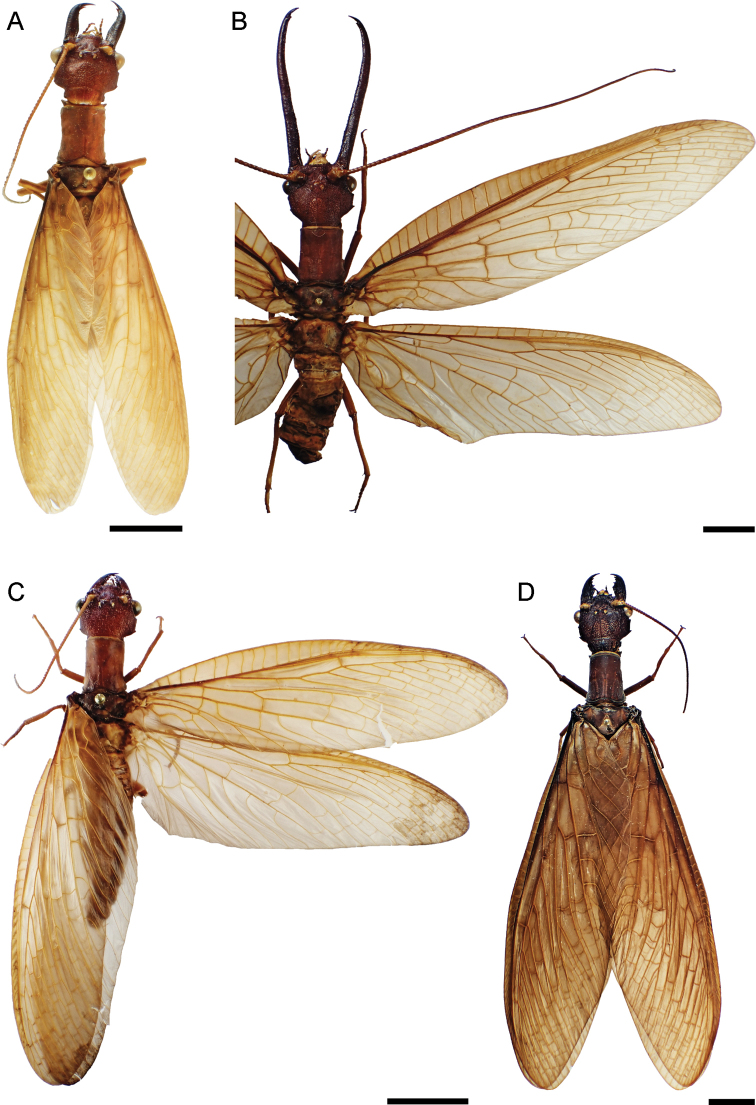
Dorsal habitus of *Corydalus* spp. **A** holotype of *C.ralphi* sp. nov., male **B** holotype of *C.wanningeri* Contreras-Ramos & von der Dunk, male **C** paratype of *C.ralphi* sp. nov., female **D** paratype of *C.wanningeri* Contreras-Ramos & von der Dunk, female. Scale bar: 1 cm.

**Figure 2.**
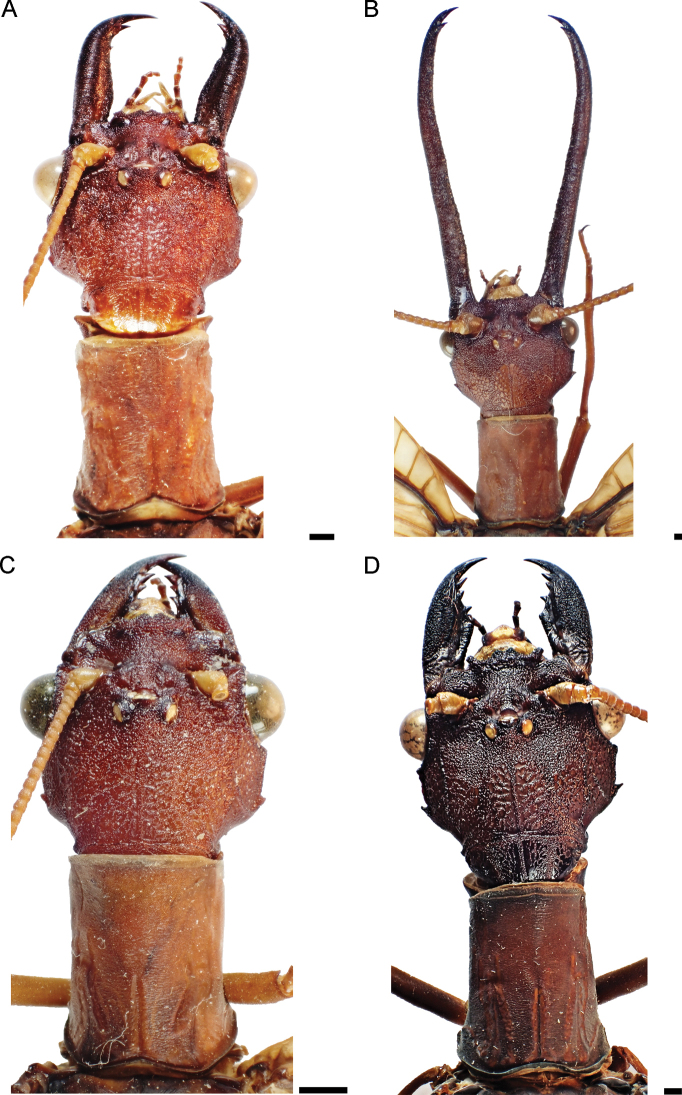
Head and pronotum of *Corydalus* spp., dorsal view. **A** holotype of *C.ralphi* sp. nov., male **B** holotype of *C.wanningeri* Contreras-Ramos & von der Dunk, male **C** paratype of *C.ralphi* sp. nov., female **D** paratype of *C.wanningeri* Contreras-Ramos & von der Dunk, female. Scale bar: 1 mm.

**Figure 3.**
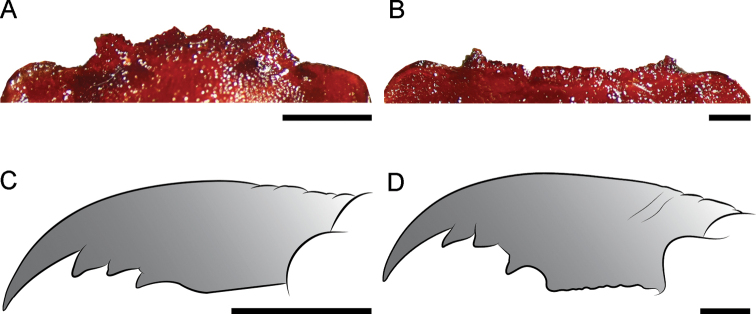
Head structures of *Corydalus* spp. **A** clypeal margin of holotype of *C.ralphi* sp. nov., male **B** clypeal margin of holotype of *C.wanningeri* Contreras-Ramos & von der Dunk, male **C** right mandible of paratype of *C.ralphi* sp. nov., female **D** right mandible of paratype of *C.wanningeri* Contreras-Ramos & von der Dunk, female. Scale bar: 1 mm.

**Figure 4.**
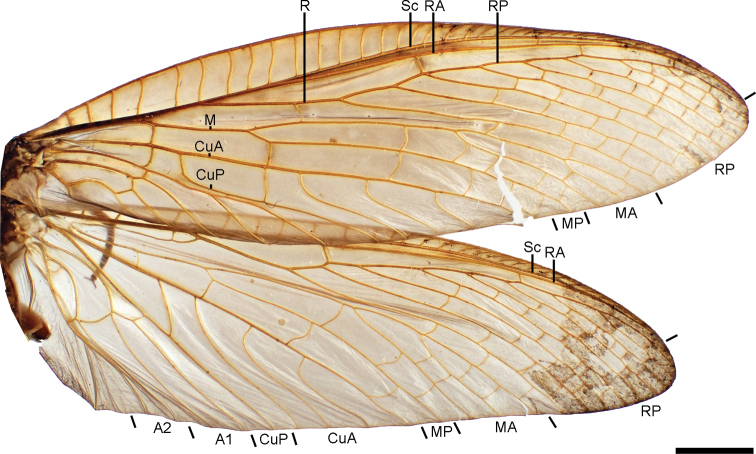
Wings of *Corydalusralphi* sp. nov. Scale bar: 5 mm. Abbreviations: A, anal veins; CuA, cubitus anterior; CuP, cubitus posterior; MA, media anterior; MP, media posterior; RA, radius anterior; RP, radial posterior; and Sc, subcosta.

**Figure 5.**
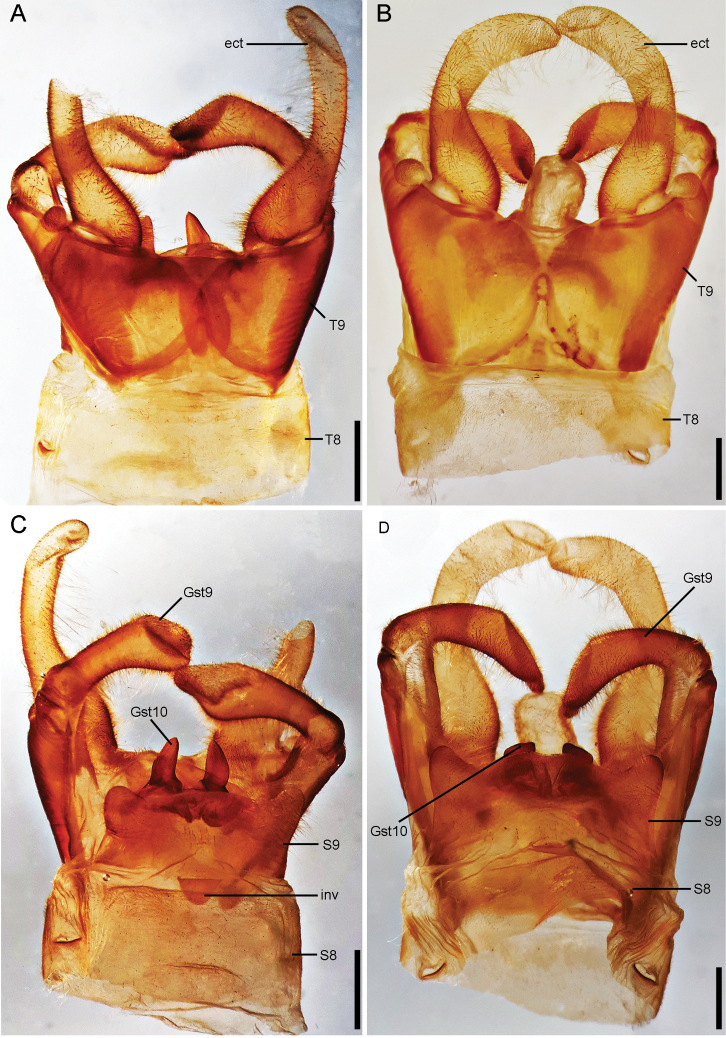
Male genitalia of *Corydalus* spp. **A** genitalia of holotype of *C.ralphi* sp. nov., dorsal view **B** genitalia of holotype of *C.wanningeri* Contreras-Ramos & von der Dunk, dorsal view **C** genitalia of holotype of *C.ralphi* sp. nov., ventral view **D** genitalia of holotype of *C.wanningeri* Contreras-Ramos & von der Dunk, ventral view. Scale bar: 1 mm. Abbreviations: ect, ectoproct; Gst 9, gonostylus 9; Gst 10, gonostylus 10; inv, invagination of membrane between segments 8 and 9; S8–9, sternites 8–9; T8–9, tergites 8–9.

**Figure 6.**
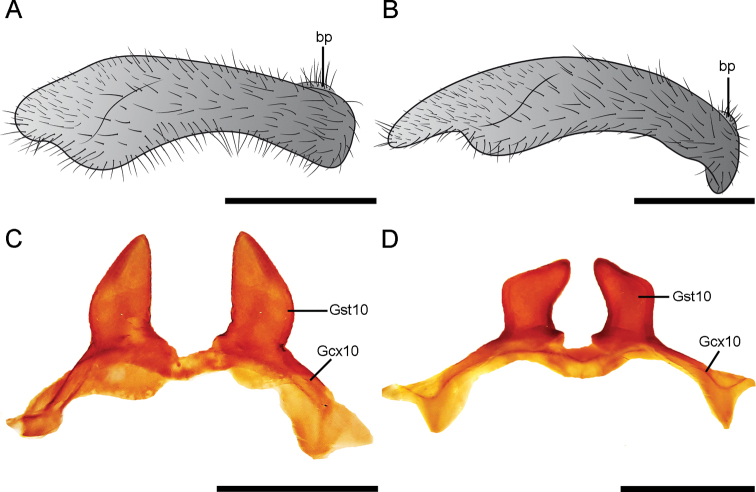
Structures of male genitalia of *Corydalus* spp. **A** gonostylus 9 of *C.ralphi* sp. nov., latero-caudal view **B** gonostylus 9 of *C.wanningeri* Contreras-Ramos & von der Dunk, latero-caudal view **C** gonocoxites and gonostyli 10 of *C.ralphi* sp. nov., ventral view **D** gonocoxites and gonostyli 10 of *C.wanningeri* Contreras-Ramos & von der Dunk, ventral view. Scale bar: 1 mm. Abbreviations: bp, basal projection of gonostylus 9; Gsx10, gonocoxite 10; Gst10, gonostylus 10.

**Figure 7.**
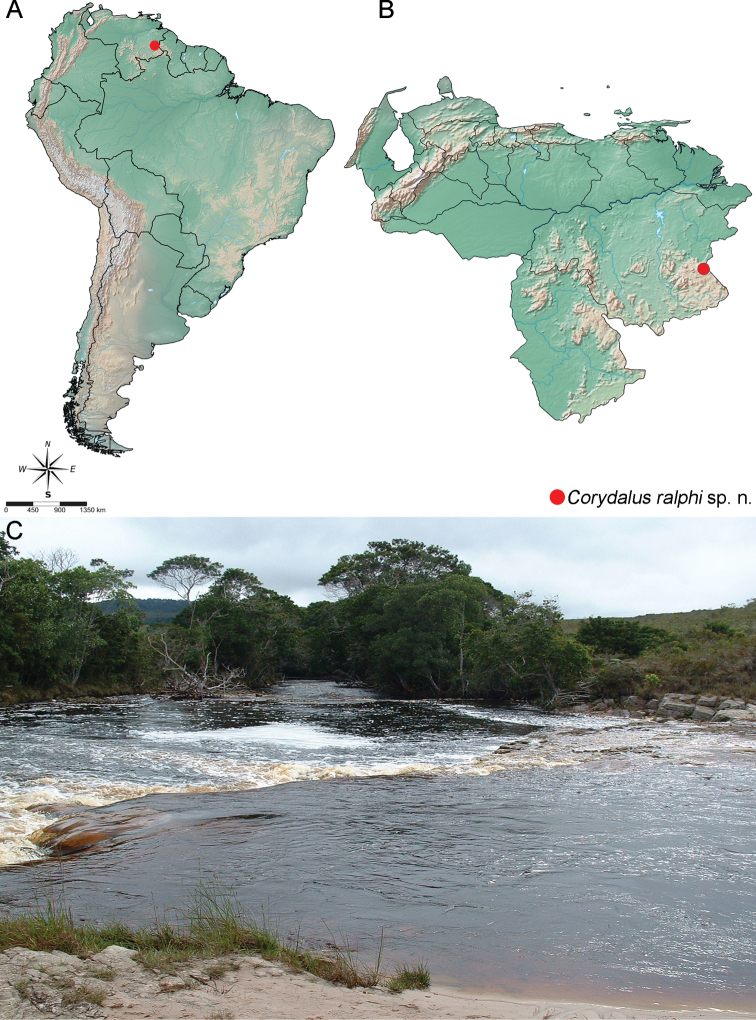
Habitat and distribution of *Corydalusralphi* sp. nov. **A** South America **B** Venezuela **C** Tarotá River, Gran Sabana region, Bolívar state, Venezuela.

Figures 1 , 2 , 3 , 4 , 5 , 6 , 7

#### Etymology.

We are glad to name this new species after Prof. Ralph W. Holzenthal of the University of Minnesota, as homage to his bright career of research and teaching, motivating several generations of new insect biodiversity professionals.

#### Type material.

***Holotype***, male, VENEZUELA: Bolívar, Parque Nacional Canaima, Gran Sabana, Río Tarotá, 5°49'15.0"N, 61°25'04.0"W, 1,324 m a.s.l., 14.iii.2007, leg. Azevedo, CAS; Hamada, N. (INPA). ***Paratype***, female, same data as holotype (INPA).

#### Diagnosis.

Head and pronotum pale reddish brown (Figs 1A, C, 2A, C), with yellowish elements on the head, especially on antennae and labrum. Body and wings generally pale reddish brown, wings unpatterned, thus resembling *C.neblinensis* Contreras-Ramos and *C.wanningeri* (Figs 1B, D, 2B, D). Male genitalia similar to *C.wanningeri*; however, in the new species the gonostylus 9 has a slightly projected and convex apex (Figs 5A, C, 6A), whereas *C.wanningeri* has a strongly extended and narrow apex (Figs 5B, D, 6B); *C.neblinensis* has a subclavate and unmodified gonostylus 9 (Contreras-Ramos 1998: fig. 26A, B). Shape of gonostyli 10 is also diagnostic. In the new species these are strongly sclerotized, almost parallel to each other, subtriangular, bluntly pointed, and caudally straight (Figs 5A, C, 6C); while they are strongly sclerotized, close to each other, convergent, and bluntly pointed in *C.wanningeri* (Figs 5B, D, 6D); and semi-membranous, widely separated, and papilliform in *C.neblinensis* (Contreras-Ramos 1998: fig. 26C). Gonostyli 10 of *C.ralphi* sp. nov. resemble those from *C.crossi*; however, the latter species is easily separated from the new one by its dark brown body and darkly patterned wings (Contreras-Ramos 2002: fig. 6). Females may be distinguished by the unpatterned pale reddish brown color (Figs 1C, 2C), and by the arrangements of the mandibular dentition (Fig. 3C), with the three basal teeth close to each other, and basal tooth smaller than the second and third ones; also with an inner predental concavity and moderately separated first and second teeth in *C.wanningeri* (Fig. 3D), and with basal tooth larger than the second and third teeth in *C.neblinensis* (Contreras-Ramos 1998: fig. 26F).

#### Description.

**Male measurements**: Head width 9.26 mm; mandible length 7.4 mm; antenna length 29.3 mm; forewing length 47.1 mm; hindwing length 40.2 mm; antenna length/forewing length 0.62. **Female measurements**: Head width 8.9 mm; mandible length 5.35 mm; antenna length 23.4 mm; forewing length 52.5 mm; hindwing length 47.7 mm; antenna length/forewing length 0.44. Body (Fig. 1A, C) pale reddish brown with yellowish elements, especially on head and thoracic pleura.

***Head*** (Figs 2A, C, 3A, C). Pale reddish brown, unpatterned. Vertex infuscated, with three yellow ocelli each surrounded by darkish brown ring. Labrum yellow, with short yellow setae. Clypeal margin darkish brown, lateral projection well developed, subtriangular; medial projection well developed, deeply incised; lateral and median projection close to each other. Male mandible elongate, pale reddish brown with margins dark brown; median tooth longer than in female and narrow; apex curved inwards, bearing three teeth, basal preapical tooth small, subtriangular, and separated from the second preapical tooth, which is closely associated with a well-developed apical tooth. Female mandible same color as male, unmodified; basal preapical tooth small, close to second and third preapical teeth; second preapical tooth smaller than third, apical tooth well developed, darkish brown. Antenna 64–66-segmented, filiform; scape yellow, subquadrangular; pedicel yellow; flagellum yellow, with apical flagellomeres darkish brown. Maxilla darkish brown to yellow; maxillar palpi 5-segmented, palpomeres pale reddish brown with yellow apex. Labium darkish brown to yellow; labial palpi 3-segmented, darkish brown to yellow.

***Thorax*** (Figs 1A, C, 2A, C). Pronotum rectangular, nearly 1.45 times longer than wide (length 7.9 mm/ width 5.4 mm), pale reddish brown, unpatterned; densely covered with minute pale brown setae. Mesonotum wider than long, pale reddish brown, unpatterned; densely covered with minute light brown setae. Metanotum similar to mesonotum, but slight narrower. Pteropleura yellow, with basal region of coxa darkish brown; covered with small yellow setae. Legs generally yellow, with small yellow setae, tarsal claws darkish brown.

***Wings*** (Fig. 4). Forewing pale reddish brown, semitranslucent, unpatterned. Venation reddish brown, darker than membrane, densely covered with minute and fine reddish brown setae. Costal field wider at the base, with several simple costal crossvein; pterostigma indistinct. Sc running parallel to RA, and fusing with its apex. Radial field with four crossveins. RP with nine branches, several crossveins present between them. Radiomedial space with four crossveins. M forked bear ¼ of the wing length; MA forked in two main branches (MA_1_ and MA_2_), MA_1_ forked near wing margin, MA_2_ unforked; MP unforked; intramedial field with five crossveins. Mediocubital space with six crossveins. Cu forked basally to M fork; CuA with four branches; CuP unforked; intracubital field with one crossvein. Cubitoanal field with two crossveins. A_1_ forked apically to Cu fork; field between A_1_ and A_2_ with one crossvein; A_2_ forked basally to A_1_ fork; field between A_2_ and A_3_ with one crossvein; A_3_ simple. Hindwing with general aspect similar to forewing. Costal field wider at the base, with several simple costal crossvein; pterostigma indistinct. Sc running parallel to RA, and fusing with its apex. Radial field with three crossveins. RP with nine branches, several crossveins present between them. Radiomedial space with five crossveins, including the elongated, sigmoid 1r-m, with a veinlet linking it to R. M forked bear ¼ of the wing length; MA forked in two main branches (MA_1_ and MA_2_), MA_1_ forked near wing margin, MA_2_ unforked; MP unforked; intramedial field with three crossveins. Cu forked near the wing base; CuA with four branches; CuP unforked; intracubital field with one crossvein. Cubitoanal field with one crossvein. A_1_ forked apically to Cu fork; field between A_1_ and A_2_ with one crossvein; A_2_ forked near the same level of A_1_ fork; field between A_2_ and A_3_ with one crossvein; A_3_ simple.

***Male genitalia*** (Figs 5, 6). Tergite 8 rectangular. Sternite 8 rectangular. Medial region of the membrane between sternites 8 and 9 presenting a large, well sclerotized, and subtriangular invagination, 1.5 times wider than long, bearing several minute setae inside and close its opening. Tergite 9 trapezoidal, cephalic V-shaped internal inflection reaching 2/3 of the length of tergite; caudally V-shaped internal inflexion reaching 1/3 of the tergite length. Anal tubercle inconspicuous. Sternite 9 subquadrate, semi-membranous, posterolateral lobes well developed. Gonostylus 9 subclavate, approximately as long as ectoproct, with apex composed by a slightly expanded and convex apex; basal protrusion present, poorly-developed. Gonocoxite 10 slightly convex, anterolateral projections well developed, wider than medial region, subtriangular; gonostylus 10 strongly sclerotized, subtriangular, almost parallel to each other, bluntly pointed, caudally straight. Ectoprocts as long as gonostylus 9, digitiform, basal 1/3 wide, roundly concave; apex slight curved inward. Pregenital sacs absent.

***Female genitalia.*** Terminalia indistinct. Sternal pouch between abdominal segments 6 and 7, abdominal segments poorly developed. Gonocoxite 8 moderately sclerotized, discontinuous with pleural area, posterior margin mesally semi-membranous, concave. Gonocoxite 9 ovoid, uniformly setose; gonostylus 9 small, semicircular; ectoproct as a small ovoid sclerite, setose.

#### Distribution (Fig. 7A, B).

Venezuela (Bolívar).

#### Habitat and bionomics (Fig. 7C).

Larvae of the new species were collected under rocks near river banks and under the roots of aquatic Cyperaceae in the Tarotá River, Gran Sabana region in Bolívar state, southern Venezuela. Adults were obtained by rearing these larvae in laboratory conditions. Tarotá River has black water and is approximately 20 m wide, with sand bottom and scattered rock and boulders.

#### Comments.

*Corydalusralphi* sp. nov. is closely related to *C.wanningeri*, both from the state of Bolívar. The new species was collected in the plateau of the Gran Sabana region, inside the Canaima National Park, whereas *C.wanningeri* was collected adjacent to the NE limit of Canaima National Park, in a portion of winding road known as La Escalera, highway 10 (connecting Orinoco lowlands with the Gran Sabana plateau in the south), which is a humid slope covered with rain forest, with several brooks and waterfalls (Contreras-Ramos and von der Dunk 2010). It is unknown whether both species are parapatric, or actually sympatric. Both species share similar coloration of wings and body (Fig. 1), nevertheless males of each species may be differentiated by the shape of the gonostylus 9 apex, slightly expanded and convex in *C.ralphi* sp. nov. (Fig. 6A) and narrow and digitiform in *C.wanningeri* (Fig. 6B); gonostyli 10 are almost parallel to each other and caudally straight in *C.ralphi* sp. nov. (Fig. 6C), and apically convergent in *C.wanningeri* (Fig. 6D); anterior margin of clypeus has medial projection well developed and deeply incised in the new species (Fig. 3A), while it is flat to slightly concave, with shallow incision in *C.wanningeri* (Fig. 3B). Other characters that help differentiate males of both species are the length of the antennae, reaching ¼ of the wing length in *C.ralphi* sp. nov. (Fig. 1A), and reaching 4/5 of the wing length in *C.wanningeri* (Fig. 1B); the new species has modified, yet short mandibles (Fig. 2A), while *C.wanningeri* has elongated mandibles (Fig. 2B); however, the variation in this trait is still unknown. Females may be separated by the dentition pattern. *Corydalusralphi* sp. nov. lacks inner predental concavity, with first and second preapical teeth close to each other (Fig. 3C), while the inner predental concavity is evident in *C.wanningeri*, as well as the first and second preapical teeth are moderately separated (Fig. 3D).

*Corydalusralphi* sp. nov. and *C.wanningeri* share a basal protrusion on male gonostylus 9 (Fig. 6A, B), as well as the general structure of gonocoxite and gonostylus 10 (Fig. 6C, D) with *C.crossi* (Contreras-Ramos 2002: figs 24, 25) (also recorded from Bolívar state), so these three species appear to be phylogenetically related. The latter species, however, may be easily separated from the former two by its darkly patterned wings.

### ﻿Key to males of *Corydalus* species from Venezuela

Modified from Contreras-Ramos (1998; 2002), and Contreras-Ramos and von der Dunk (2010)

**Table:** 

1	Abdomen: ectoproct short and broad (Contreras-Ramos 1998: fig. 5A), or tubular and sharply bent (Contreras-Ramos 2002: fig. 34); gonostylus 10 reduced, inconspicuous (Contreras-Ramos 1998: fig. 18C; Contreras-Ramos 2002: fig. 36)	**2**
–	Abdomen: ectoproct elongate, tubular (Fig. 5; Contreras-Ramos 2002: fig. 16); gonostylus 10 conspicuous, well developed (Fig. 6C, D; Contreras-Ramos 1998: fig. 4C)	**5**
2	Head: strongly patterned with brownish and yellowish areas (Contreras-Ramos 2011: fig. 3C); Forewing: semitranslucent, conspicuously spotted (Contreras-Ramos 2011: fig. 3C); Abdomen: ectoproct broadly conical (Contreras-Ramos 1998: fig. 18A)	***C.flinti* Contreras-Ramos (Venezuela)**
–	Head: unpatterned (Contreras-Ramos 2011: fig. 3B); Forewing: not so translucent, neither spotted (Contreras-Ramos 2011: fig. 3B); Abdomen: ectoproct shaped otherwise, flattened (Contreras-Ramos 1998: fig. 5A, E) or variously curved (Contreras-Ramos 2002: figs 28, 34)	**3**
3	Head: postocular spine well developed (Contreras-Ramos 2011: fig. 3B); Abdomen: gonostylus 9 with apex narrow, directed dorsally, portion of internal apodeme as external outgrowth (Contreras-Ramos 1998: fig. 5A, E); ectoproct somewhat flattened, with dorsal elongate process (Contreras-Ramos 1998: fig. 5A, B)	***C.arpi* Navás (Brazil, Venezuela)**
–	Head: postocular spine slightly developed (Contreras-Ramos 2002: figs 7, 8); Abdomen: gonostylus 9 with apex blunt or uniformly tubular (Contreras-Ramos 2002: figs 29, 35), outgrowth of apodeme absent; ectoproct with different shape	**4**
4	Abdomen: gonostylus 9 subclavate (Contreras-Ramos 2002: figs 28, 29); sternite 9 with conspicuous sclerotized median projection (Contreras-Ramos 2002: fig. 29); ectoproct strongly curved, simple (Contreras-Ramos 2002: fig. 28)	***C.hayashii* Contreras-Ramos (Venezuela)**
–	Abdomen: gonostylus 9 uniformly tubular (Contreras-Ramos 2002: figs 34, 35); sternite 9 with internal sclerotized ridge but lacking median projection (Contreras-Ramos 2002: fig. 35); ectoproct strongly curved (Contreras-Ramos 2002: fig. 34), with broad projection directed ventrally (Contreras-Ramos 2002: figs 35, 37)	***C.mayri* Contreras-Ramos (Venezuela)**
5	Abdomen: sternite 9 modified, with posteromedian projection (Contreras-Ramos 2002: fig. 17) or sub-attenuate and more sclerotized posteromedially (Contreras-Ramos 1998: fig. 27B)	**6**
–	Abdomen: sternite 9 unmodified, subquadrate (Fig. 5A, B; Contreras-Ramos 1998: figs 2B, 4B)	**8**
6	Abdomen: sternite 9 sub-attenuate, noticeably more sclerotized posteromedially (Contreras-Ramos 1998: fig. 27B)	***C.nubilus* Erichson (Brazil, Colombia, French Guiana, Guyana, Venezuela)**
–	Abdomen: sternite 9 with posteromedian projection (Contreras-Ramos 2002: fig. 17)	**7**
7	Abdomen: posteromedian projection of sternite 9 large (nearly as long as sternum), thumblike (Contreras-Ramos 1998: fig. 31B); gonostylus 9 unguiform (Contreras-Ramos 1998: fig. 31B)	***C.tesselatus* Stitz (Colombia, Venezuela)**
–	Abdomen: posteromedian projection of sternite 9 small (~ 1/2 as long as sternum), narrow (Contreras-Ramos 2002: fig. 17); gonostylus 9 tubular (Contreras-Ramos 2002: fig. 17)	***C.clavijoi* Contreras-Ramos (Venezuela)**
8	Abdomen: gonostylus 9 elongate, somewhat flattened or tubular (Contreras-Ramos 1998: figs 2B, 19B)	**9**
–	Abdomen: gonostylus 9 subclavate (Fig. 6A, B; Contreras-Ramos 1998: figs 4B, 7B, 17B)	**11**
9	Abdomen: gonostylus 9 narrower and noticeably shorter than ectoproct (Contreras-Ramos 2002: fig. 23)	***C.crossi* Contreras-Ramos (Venezuela)**
–	Abdomen: gonostylus 9 and ectoproct subequal in length and shape (Contreras-Ramos 1998: figs 2A, 19A)	**10**
10	Abdomen: gonostylus 9 somewhat flattened (Contreras-Ramos 1998: fig. 2A, B), ectoproct base as wide as median region (Contreras-Ramos 1998: fig. 2A, B)	***C.affinis* Burmeister (Argentina, Bolivia, Brazil, Colombia, Ecuador, French Guiana, Guyana, Paraguay, Peru, Venezuela)**
–	Abdomen: gonostylus 9 tubular (Contreras-Ramos 1998: fig. 19A, B); ectoproct base wider than median region (Contreras-Ramos 1998: fig. 19A, B)	***C.hecate* (McLachlan) (Brazil, Peru (?), Venezuela(?))**
11	Head: reddish brown (Fig. 2); Thorax: pronotum reddish brown (Fig. 2); Abdomen: ectoproct apex without incurvation (Fig. 5B, D; Contreras-Ramos and von der Dunk 2010: fig. 5) or slightly curved, although it may be enlarged (Fig. 5A, C; Contreras-Ramos 1998: figs 7F, 26E)	**12**
–	Head: yellowish to greenish brown; Thorax: pronotum yellowish to greenish brown; Abdomen: ectoproct apex with well-developed incurvation (Contreras-Ramos 1998: figs 4A, 17B)	**15**
12	Forewing: contrastingly patterned (Contreras-Ramos 1998: fig. 58)	***C.batesii* McLachlan (Bolivia, Brazil, Colombia, Ecuador, French Guiana, Guyana, Peru, Suriname, Venezuela)**
–	Forewing: not so contrastingly patterned (Fig. 1)	**13**
13	Forewing: pale, clear, nearly translucent, few subtle small white spots (Contreras-Ramos 1998: figs 124–126); Abdomen: gonostylus 9 unmodified (Contreras-Ramos 1998: fig. 26B; gonostylus 10 papilliform (Contreras-Ramos 1998: fig. 26C)	***C.neblinensis* Contreras-Ramos (Venezuela)**
–	Forewing: rather opaque, uniformly pale reddish (Fig. 1; Contreras-Ramos and von der Dunk 2010: fig. 1); Abdomen: gonostylus 9 with expanded apex (Fig. 6A, B; Contreras-Ramos and von der Dunk 2010: figs 5, 6); gonostylus 10 elongate-trianguloid (Fig. 6C, D; Contreras-Ramos and von der Dunk 2010: figs 5, 6)	**14**
14	Head: anterior margin of clypeus with medial projection well developed and deeply incised (Fig. 3A); Abdomen: gonostylus 9 with slightly expanded and convex apex (Fig. 6A); gonostyli 10 almost parallel (Fig. 6C)	***C.ralphi* sp. nov. (Venezuela)**
–	Head: anterior margin of clypeus with flat to slightly concave, with shallow incision (Fig. 3B); Abdomen: gonostylus 9 with strongly expanded and narrow apex (Fig. 6B); gonostyli 10 convergent (Fig. 6B)	***C.wanningeri* Contreras-Ramos & von der Dunk (Venezuela)**
15	Head: antenna conspicuously subserrate, sinuate (Contreras-Ramos 1998: fig. 17F); Abdomen: gonocoxites 10 with anteromedian projection (Contreras-Ramos 1998: fig. 17C)	***C.flavicornis* Stitz (Brazil, Colombia, Costa Rica, Ecuador, El Salvador, Guatemala, Honduras, Panama, Peru, Venezuela)**
–	Head: antenna slightly subserrate; Abdomen: gonocoxites 10 without anteromedian projection (Contreras-Ramos 1998: fig. 4C)	**16**
16	Head: antenna, including scape and pedicel, pale to dark brown, apically infuscate (Contreras-Ramos 1998: figs 43, 44, 48); Abdomen: gonostyli 10 lobes typically subequal in width and length, less than half length of lobe surpassing posterior edge of gonocoxites 10 (Contreras-Ramos 1998: fig. 4C); pregenital sacs well developed, conspicuous (Contreras-Ramos 1998: fig. 4F)	***C.armatus* Hagen (Argentina, Bolivia, Brazil, Colombia, Ecuador, Peru, Venezuela)**
–	Head: antenna, including scape and pedicel, yellow to yellowish green, up to distal 1/3 infuscate (Contreras-Ramos 1998: figs 139–141); Abdomen: gonostyli 10 typically ~ 2× as long as wide, ~ 1/2× lobe surpassing posterior edge of gonocoxites 10 (Contreras-Ramos 1998: fig. 29C); pregenital sacs apparently absent, inconspicuous	***C.peruvianus* Davis (Argentina, Bolivia, Brazil, Colombia, Costa Rica, Ecuador, Guatemala, Honduras, Mexico, Nicaragua, Panama, Peru, Venezuela)**

## Supplementary Material

XML Treatment for Corydalus ralphi
